# Histopathological observation and *HSP* genes expression analysis of *Puccinia triticina* under high-temperature stress in Jinmai region

**DOI:** 10.3389/fmicb.2025.1546550

**Published:** 2025-03-27

**Authors:** Yaqiong Zhao, Yashi Guan, Miaomiao Liu, Weiwei Gong, Fuxin He, Minjie Liu

**Affiliations:** College of Plant Protection, Shanxi Agricultural University, Taiyuan, China

**Keywords:** *Puccinia triticina*, temperature sensitivity, histopathology, infection process, heat shock protein

## Abstract

**Introduction:**

Wheat leaf rust is one of the most significant diseases affecting wheat. With the increase of global average temperature, the areas where wheat leaf rust can survive winter and persist through summer have been expanding. And high-temperature resistant isolates were identified within the natural population.

**Methods:**

In this study, two high-temperature resistant strains and two temperature-sensitive strains were selected from 41 isolates collected from Shanxi Province between 2021 and 2022. The analysis of disease severity differences among the four strains at different temperatures revealed that the differences were most significant at 26°C (*p* < 0.05). Therefore 26°C was selected as the optimal temperature for high-temperature stress. Subsequently, histopathological observations were conducted on the four *Pt* strains, and the expression levels of five genes were determined, including *PtHsp90-1*, *PtHp*, *PtHspHSS1*, *PtHsp60* and *PtHspSSB*.

**Results:**

Histopathological observations showed that compared with the temperature-sensitive strains, the number of haustorial mother cells and haustoria of high-temperature resistant was not significantly affected by high-temperature stress (*p* < 0.05). However, the number of hyphal branches of four strains was less affected, indicating that formation of haustorial mother cells and haustoria were the key stages in resistance to heat stress. Further analysis revealed that the expression of five genes reached their maximum at 6 hpi under high-temperature stress. The results indicated that *HSP* genes play a crucial role in high-temperature resistance during the germination stage of *Pt* urediospores.

**Discussion:**

However, the specific molecular mechanism in wheat leaf rust required further study and verification. In conclusion, the early germination of urediospores (0–12 h) and the formation of haustorial mother cells and haustoria (12–24 h) were key stages in resisting high-temperature stress in wheat leaf rust.

## 1 Introduction

Wheat leaf rust, caused by *Puccinia triticina* (*Pt*), is a significant airborne disease affecting wheat production globally. Severe infections can result in yield losses exceeding 50% (Ordoñez et al., 2010). Currently, planting resistant varieties was the most economical and effective measure to control wheat leaf rust. However, these resistant varieties often lose resistance due to the continuous emergence and evolution of new pathotypes of the disease. The Northwest, Huang-huai-hai and the middle and lower reaches of Yangtze River were the oversummering areas of *Pt*. The virulence and pathogenicity structures of pathogen populations in these oversummer area remained unclear. And with the increase of global average temperature, plant pathogens have also undergone various adaptations to cope with environmental changes (Ghelardini et al., 2016). *Pt* urediospores exhibit a wide temperature adaptability, capable of germinating at temperatures ranging from 2 to 31°C, demonstrating both low and high-temperature tolerance (Thistlethwaite et al., 2020). The areas where *Pt* can survive winter and persist through summer have expanded significantly. In the middle and lower reaches of the Yangtze River, when winters were warm and humid, wheat leaf rust can not only overwinter but also expand on wheat to some extent, providing a substantial number of urediospores for the spread and prevalence of leaf rust in the following spring. Temperature was the most critical environmental factor influencing the spread of leaf rust into wheat tissue. Therefore, it is urgent to conduct research on the impact of temperature on the prevalence of wheat leaf rust and the molecular mechanism of high-temperature resistance.

The IPCC has used global temperature records and climate models to assess warming trends, noting an increase of 0.6°C from 1901–2001 (David and Maria, 2002), 0.74°C from 1906–2005 (IPCC, 2007), and 0.85°C from 1880–2012 (IPCC, 2013). Global warming has shifted crop planting zones northward, along with the boundaries for pests and diseases. Studies have shown that rising temperatures can accelerate the life cycles of pathogens and pests, such as wheat stripe rust (Lian et al., 2016) and powdery mildew (zhang et al., 2022), with significant impacts on disease prevalence. For example, at 5.5–8.6°C, the incubation period for wheat leaf rust is 22–30 days, but only 5 days at 25°C. Research also indicated that pathogen virulence correlates with temperature sensitivity and latitude (Bebber et al., 2013; Bebber, 2015). In China, increasing temperatures in wheat-growing regions have facilitated the spread of leaf rust, prompting development of high-temperature resistant strains. However, the mechanisms behind this resistance require further investigation to mitigate future disease risks effectively.

At present, the understanding of the high-temperature resistance mechanism in fungi primarily came from model strains such as *Saccharomyces cerevisiae* and *Neurospora crassus* (Shin et al., 2018; Zhou et al., 2018). Studies have shown that there were three main mechanisms by which yeasts respond to heat stress. First, heat stress leaded to the synthesis of a large number of heat shock proteins (HSPs). HSPs promoted the correct refolding of proteins that misfold at high temperatures, inhibited the accumulation of misfolded proteins, and prevented protein inactivation. Consequently, cellular damage from heat stress damage can be avoided or reduced, and physiology recovery after stress can be facilitated (Malini et al., 2020). Second, a high concentration of trehalose in cytoplasm can prevent denaturation and inactivation of enzymes and proteins under stress conditions. Trehalose can reduce protein aggregation, stabilize cells, increase trehalose accumulation. This also helped to maintain the stability of the cell membrane and protected cellular DNA and lipids from stress damage (Levin, 2011). Third, the hypertonic glycerol (HOG) signaling pathway and cell wall integrity (CWI) signaling pathway were activated. These pathways protect cells from stress by activating a series of downstream effector genes (Lodder et al., 1999; Verna et al., 1997). Heat shock proteins were among the proteins most closely related to biological heat resistance (Liu et al., 2019). HSPs were present in all somatic cells of prokaryotes and eukaryotes, usually functioning as molecular chaperone, and their expression increaseed sharply when the organism was subjected to high temperature stress (Vabulas et al., 2010).

HSPs prevented misfolding or random aggregation of proteins by binding to reactive active regions of proteins. It can prevent the protein denaturation and reduce the adverse effects of heat stress on the growth and development of organism (zhang et al., 2022). With the development of molecular techniques, it is now possible to conduct GWAS analysis by combining gene sequencing (Chu et al., 2024), molecular markers, and biological phenotype data (Huang et al., 2022), enabling the rapid identification of relevant genes. To study the cellur and molecular mechanism of wheat leaf rust to response to temperature, histopathology and molecular biology methods were used to screen heat-tolerant genes and to clarify their expression patterns and gene functions. Based on previous studies, we collected samples of wheat leaf rust from different cities and counties in Shanxi Province in 2021 and 2022. The temperature sensitivity of the isolates was determined, and different temperature sensitive strains were selected based to the ET_50_ value. The germination rate of urediospores and the histological structure of the selected strains were observed under high-temperature treatment.

## 2 Materials and methods

### 2.1 Materials

In this study, the highly susceptible winter wheat cultivar mingxian 169 was utilized for propagating urediospores of *Pt*. To evalutae the virulence profiles of the isolates Pt strains, a set of near-isogenic wheat lines derived from Thatcher were employed. These lines included: TcLr1, TcLr2A, TcLr2C, TcLr3, TcLr9, TcLr16, TcLr24, TcLr26, TcLr3kA, TcLr11, TcLr17, TcLr30, TcLrB, TcLr10, TcLr14A, TcLr18. The near-isogenic lines were provided by the Institute of Plant Protection, Chinese Academy of Agricultural Sciences and Institute of Plant Protection, Gansu Academy of Agricultural Sciences. The *Pt* samples were collected from the susceptible areas in Shanxi province in 2021 and 2022. Diseased leaves were wrapped in absorbent paper and placed in envelopes, which were labeled with the cultivar name, sampling location, date of collection, and geographical coordinates including longitude, latitude and altitude.

### 2.2 Isolation and propagation of *Pt* strains

The wheat samples collected in the filed were numbered according to the cities and counties of origin, then separated accordingly. A 9 cm diameter Petri dish was prepared with filter paper moistened using sterilized distilled water. Fresh samples were spread on the filter paper, the dish was covered, and then placed at 4°C. Single urediospore isolation was conducted after 12 h of dark and humid conditions. Samples containing fresh urediospores were placed in the culture dish at 4°C in darkness for 12 h prior to the isolation of single urediospore (Zhao et al., 2019). Mingxian 169 was sown in pots approximately 7 cm in diameter (about 10 grains per pot) and placed in an artificial climate with the following environmental parameters: 16 h light at 10,000 lux intensity, temperature maintained between 18–20°C, and humidity kept at 60–70%. The plants were grown until the first leaf was fully deployed for *Pt* isolation. Fresh urediospores from the leaves were carefully collected using an inoculation needle and numbered. After about 7 days, the infected urediospores were transferred to healthy Mingxian 169 to propagate them to sufficient quantities (Zhang et al., 2015).

### 2.3 Determination of temperature sensitivity of *Pt* strains

The isolates of *Pt* were inoculated onto the highly susceptible variety Mingxian 169, and then placed in light incubators set at different temperature: 18°C, 20°C, 22°C, 24°C, 26°C and 28°C, respectively, (environmental parameters: 16 h light time, 10,000 lux light intensity, 60–70% humidity). The 18°C condition served as the control, with each temperature treatment repeated three times. Approximately 10 days post-inoculation (dpi), the disease severity of different strains under various temperature treatments was assessed, and the disease inhibition rate was calculated. According to DB11-T283-2005 wheat leaf rust survey standards, the average severity was calculated using the following Equation 1:


(1) $$D=\frac{\sum \left(d_{i}\times l_{i}\right)}{L}\times 100$$

In which *D* is the average severity (%), *d_i_* is the level i severity value, *l_i_* is the number of diseased leaves of grade i. L is the total number of diseased leaves investigated, i is 1, 5, 10, 20, 40, 60, 80 and 100%. Disease inhibition rate using the following Equation 2:


(2) $$y=\left(1-\frac{D_{t}}{D_{c}}\right)\times 100$$

In which *Y* is disease inhibition rate (%), *D_t_* is the average severity of treatment group, *D_c_* is the average severity of control group. The regression equation was established using disease inhibition rate (*Y*) and temperature (*X*), the Equation 3 is as follow:


(3) Y=aX+b

The value of *X* was calculated as the median disease inhibition temperature (ET_50_) when *Y* was 50% (Han et al., 2016). Based on the ET_50_ values, the strain corresponding to the maximum value was selected as the high-temperature resistant strain, and the strain corresponding to the minimum value was selected as the temperature-sensitive resistant strain. Statistical analysis of the results was conducted using SPSS (Statistical Product and Service Solutions, version 21.0, IBM, American), and multiple comparisons were performed using Duncan’s test at the *p* = 0.05 significance level. The bar charts in the paper were created using Origin 2024 software.

### 2.4 Determination of urediospores germination rate, virulence identification and histopathological observation of *Pt* strains

1 mg of fresh urediospores was evenly dispersed on the surface of 15 cm petri dish containing 0.05% water agar. The urediospores were incubated in darkness at five different temperatures: 15°C (control), 20°C, 25°C, 30°C and 35°C. After approximately 24 h, germination was observed under an optical microscope. Germination was defined as the length of the germ tube exceeding half of the diameter of the urediospores. The number of germinated and ungerminated urediospores was counted to calculate the germination rate. For each temperature treatment, at least 300 urediospores were observed, and the experiment was repeated to ensure reliability.

A suspension of urediospores was prepared by mixing 10 mg urediospores with 20 mL 0.2% Tween 80 solution. This suspension was used to inoculated 16 wheat near-isogenic lines and the susceptible variety Mingxian 169. The infection types was recorded according to the Roelfs (1985) standard, and the strains were named using the cryptographic naming system outlined by Long and Kolmer (1989).

In this study, the infection process of wheat leaf rust was observed using a living leaf transparency technique combined with WGA-Alexa488 fluorescent dye. First, wheat leaves about 2 cm were placed in 40 mL fixing solution (95% ethanol:glacial acetic acid = 3:1), and the of the leaves basically faded after 24 h. Then the fixed leaves were removed with tweezers and put them in a transparent liquid (25 g chloral hydrate dissolved in 10 mL distilled water) for 24 h until the leaves were completely transparent. The treated wheat leaves were stained with WGA-Alexa488 fluorescent dye. The specific steps were as follows: the fixed transparent leaves were washed with 50% ethanol for 15 min, repeated twice, and then washed with sterilized distilled water for 10 min, repeated twice. The leaves were sandwiched into a sterilized 2 mL centrifuge tube with tweezers and 1.0 M KaOH solution was added. Then it was sterilized at 121°C for 3–5 min or bathed in boiling water for 20–30 min to soften the leaf tissue and make it easy for fluorescent dye to enter the leaf cells, which was washed with distilled water for 10 min. Tris–HCl buffer (50 mM, pH = 7.0–7.4) was added into the centrifuge tube for 30 min, and then 20 μg/mL WGA-Alexa488 fluorescent dye was added for light shielding for more than 10 min. After dyeing, the leaves were rinsed with distilled water for 10 min, repeated 2–3 times, and stored in 50% glycerin for subsequent microscopic observation. The fluorescence microscope OLYMPUS BX53 was used to observe and count the number of mycelium branches, haustorium mother cells (HMC) and haustorium (H). At least 20 infection sites were observed for each treatment and the entire process was repeated three times to ensure reliability.

### 2.5 Differential gene expression of high-temperature resistant and temperature-sensitive strains under high temperature stress

Total RNAfrom *Pt* urediospores was extracted using the Ultrapure RNA Kit (Cwbio, Beijing). First-strand cDNA synthesis was performed using MonAmp™ ChemoHS qPCR Mix (Monad, Suzhou), and real-time fluorescence quantitative qRT-PCR was conducted using the PrimeScript™ RT Master Mix (Takara, Dalian). Primers for detecting the relative expression levels of differential expressed gene using NCBI Primer-BLAST and were listed in Supplementary Table 1. The *Pt* gene (GeneBank accession No. XM_053160605) served as the reference gene. The qRT-PCR reaction conditions were as follows: pre-denaturation 95°C for 30 s, 40 cycles of 95°C for 10 s, 55°C for 10 s, 72°C for 30 s, and the experiment was repeated three biological replicates. The relative gene expression levels were calculated using the 2^-ΔΔCt^ method (Livak and Schmittgen, 2001).

## 3 Result

### 3.1 Isolation of *Pt* strains from different sampling sites

The standard samples collected in 2021 and 2022 were isolated using the method described in section 2.2, yielding a total of 41 isolated strains. Specifically, the distribution of these isolates across different regions was as follows: 8, 7, 10, 5 and 11 strains from Jinzhong, Linfen, Changzhi, Jincheng and Yuncheng, respectively (Table 1).

**Table 1:** Collection information of *Pt* strains.

City	Country (Code)	NO. of isolates	Latitude (°)	Longitude (°)	Elevation (m)	Year
Jinzhong(K)	Yuci(yu)	6	37.5415	112.6773	804	2022
	Taigu(tai)	2	37.4211	112.5512	789	2021
Linfen(L)	yaodu(yao)	4	36.0830	111.5787	470	2021,2022
	Qumo(qu)	2	35.6672	111.5192	492	2021
	Yicheng(yi)	1	35.7382	111.7183	589	2022
Changzhi(D)	Lucheng(lu)	4	36.2139	113.1457	932	2021,2022
	Tunliu(tun)	5	36.3040	112.9374	906	2021
	Licheng(li)	1	36.5022	113.3872	728	2022
Jincheng(E)	Gaoping(gao)	4	35.7980	112.9235	833	2022
	Zezhou(ze)	1	35.5974	112.9573	750	2021
Yuncheng(M)	Yanhu(yan)	4	35.0150	110.9981	370	2021
	Jiang(jiang)	5	35.6162	111.2247	397	2021,2022
	Jishan(ji)	2	35.6040	110.9831	387	2022

### 3.2 Determination of temperature sensitivity of isolated strains

The temperature sensitivity of 41 isolated strains was determined by culturing them at the temperatures of 18°C, 20°C, 22°C, 24°C, 26°C and 28°C, respectively. Disease severity and inhibition medium temperature (ET_50_) were measured for each strain. Linear regression analysis was performed and the regression equation were established (Tables 2, 3). As shown in the tables, as the treatment temperature gradually increased, the disease severity of the 41 strains generally exhibited a decreasing trend, consistent with linear regression. Specially: In 2021, the lowest ET_50_ value was 18.78°C for strain 21-M-jiang-30, and the highest ET_50_ value was 25.39°C for strain 21-M-yan-1. In 2022, the lowest ET_50_ value was 19.92°C for strain 22-M-jiang-5, and the highest ET_50_ value was 25.50°C for strain 22-M-ji-3. These results highlight the varying temperature sensitivities among the isolated strains. Based on these findings, strains 21-M-jiang-30 and 22-M-jiang-5 were designated as temperature-sensitive strains S1 and S2, respectively. Conversely, strains 21-M-yan-1 and 22-M-ji-3 were classified as high-temperature resistant strains R1 and R2. These four strains were selected for subsequent analyses of biological characteristics and gene expression under high temperature stress.

**Table 2:** Determination of ET_50_ values of strains of *Pt* at different temperatures.

Number	Strain number	Disease severity (%)						ET_50_	Fitting line	R^2^
		18	20	22	24	26	28			
1	21-D-tun-5	91.72	77.74	55.26	52.54	23.61	9.64	23.23 ± 0.14	y = 5.97–0.26x	0.967
2	21-D-tun-6	83.25	44.14	47.64	42.79	26.3	16.01	21.78 ± 0.28	y = 3.61–0.16x	0.798
3	21-D-tun-25	74.68	64.65	52.46	40.00	37.59	30.47	23.00 ± 0.06	y = 2.73–0.12x	0.913
4	21-D-tun-26	94.33	81.84	46.89	51.21	27.05	8.07	23.23 ± 0.15	y = 6.47–0.28x	0.940
5	21-D-tun-27	92.14	63.56	63.00	44.33	44.41	11.44	23.49 ± 0.15	y = 5.06–0.22x	0.870
6	21-D-lu-1	91.35	67.75	45.19	48.00	33.52	22.28	23.26 ± 0.28	y = 4.53–0.19x	0.844
**7**	21-D-lu-5	69.07	75.83	48.26	45.55	36.61	4.97	22.39 ± 0.21	y = 4.48–0.20x	0.777
8	21-D-lu-7	70.83	62.44	45.59	43.09	39.55	7.35	22.01 ± 0.64	y = 3.70–0.17x	0.802
9	22-D-li-1	70.66	77.55	50.63	49.76	9.78	6.17	22.08 ± 0.11	y = 5.25–0.24x	0.815
10	22-D-lu-1	91.11	93.00	65.15	44.92	11.98	9.71	23.33 ± 0.16	y = 7.18–0.31x	0.903
11	21-M-jiang-29	76.28	77.76	50.92	51.79	49.60	15.20	23.66 ± 0.45	y = 3.71–0.16x	0.798
12	21-M-jiang-30	52.83	43.89	44.03	15.14	28.61	11.69	18.78 ± 1.05	y = 2.28–0.12x	0.724
13	21-M-yan-1	95.49	76.04	50.32	65.48	38.65	45.05	25.39 ± 0.47	y = 4.28–0.17x	0.713
14	21-M-yan-2	80.56	66.45	51.32	39.58	31.11	9.17	22.41 ± 0.22	y = 4.52–0.20x	0.942
15	21-M-yan-3	97.80	58.64	53.97	28.42	42.78	2.31	22.65 ± 0.11	y = 7.23–0.32x	0.807
16	21-M-yan-35	81.27	76.85	44.01	50.24	31.48	37.52	23.73 ± 0.69	y = 3.28–0.14x	0.759
17	22-M-jiang-5	49.97	59.90	51.17	16.07	10.00	8.82	19.92 ± 0.34	y = 3.53–0.18x	0.780
18	22-M-jiang-7	93.77	96.46	87.35	74.01	26.03	5.02	24.63 ± 0.08	y = 8.41–0.35x	0.874
19	22-M-jiang-13	99.99	96.12	87.43	81.42	6.86	7.17	24.55 ± 0.03	y = 11.19–0.46x	0.866
20	22-M-ji-3	95.77	87.46	97.17	73.38	53.63	8.83	25.50 ± 0.13	y = 6.94–0.27x	0.765
21	22-M-ji-4	87.41	75.93	67.63	41.71	43.35	3.61	23.43 ± 0.10	y = 6.01–0.26x	0.840
22	21-E-ze-2	96.32	72.44	63.69	43.18	24.14	9.07	23.21 ± 0.21	y = 6.71–0.29x	0.958
23	22-E-gao-2	97.96	57.71	41.28	37.03	25.41	5.57	22.24 ± 0.34	y = 6.13–0.27x	0.816
24	22-E-gao-4	67.81	83.03	84.63	40.75	23.70	5.39	23.04 ± 0.07	y = 5.55–0.24x	0.708
25	22-E-gao-5	80.60	96.11	62.17	49.70	12.81	7.14	23.13 ± 0.14	y = 6.92–0.30x	0.806
26	22-E-gao-6	97.14	59.20	36.11	41.49	18.48	6.10	22.10 ± 0.14	y = 5.94–0.27x	0.861
27	21-L-qu-20	96.55	75.38	58.33	51.70	17.30	11.38	23.25 ± 0.16	y = 6.78–0.29x	0.945
28	21-L-qu-21	99.33	77.78	63.33	60.76	44.99	20.44	24.69 ± 0.47	y = 6.20–0.25x	0.844
29	21-L-yao-1	57.48	56.24	42.89	32.96	30.79	1.67	20.32 ± 1.21	y = 4.12–0.20x	0.708
30	22-L-yao-1	81.91	82.34	48.01	42.78	11.90	10.67	22.47 ± 0.19	y = 5.63–0.25x	0.892
31	22-L-yao-5	56.31	52.31	37.44	46.71	12.57	7.73	20.15 ± 0.16	y = 3.27–0.16x	0.808
32	22-L-yao-7	78.80	59.44	81.96	57.95	17.93	6.60	22.99 ± 0.19	y = 5.25–0.23x	0.718
33	22-L-yi-1	81.73	88.86	76.14	67.26	25.73	6.93	23.99 ± 0.46	y = 6.17–0.26x	0.753
34	21-K-tai-1	87.17	59.97	53.13	55.38	32.71	11.65	22.99 ± 0.22	y = 4.55–0.20x	0.870
35	21-K-tai-2	81.78	49.20	47.38	34.74	14.32	15.46	21.39 ± 0.56	y = 4.09–0.19x	0.869
36	22-K-yu-1	97.34	76.67	60.95	40.00	11.35	4.67	22.81 ± 0.17	y = 8.10–0.35x	0.959
37	22-K-yu-2	90.20	91.11	61.44	46.11	12.78	12.87	23.32 ± 0.14	y = 7.04–0.30x	0.875
38	22-K-yu-7	86.35	81.60	57.78	54.52	13.24	6.72	22.96 ± 0.03	y = 6.32–0.28x	0.925
39	22-K-yu-11	81.12	82.55	70.00	20.85	14.89	4.64	22.39 ± 0.13	y = 6.51–0.29x	0.866
40	22-K-yu-12	81.42	71.30	65.00	70.47	9.47	5.60	22.99 ± 0.19	y = 5.84–0.26x	0.743
41	22-K-yu-15	96.21	85.56	59.26	72.40	22.98	6.22	23.88 ± 0.18	y = 7.59–0.32x	0.868

**Table 3:** Comparison of ET_50_ of isolates of wheat leaf rust in different regions.

City	NO. of isolates	ET_50_			
		Mean value	Minimum value	Maximum value	Maximum difference
Changzhi	10	22.78	21.78	23.49	1.71
Yuncheng	11	23.15	18.78	25.50	6.72
Jincheng	5	22.74	22.10	23.21	1.11
Linfen	7	22.55	20.15	24.69	4.54
Jinzhong	8	22.84	21.39	23.88	2.49
Total	41	22.81	–	–	–

**Figure 1:**
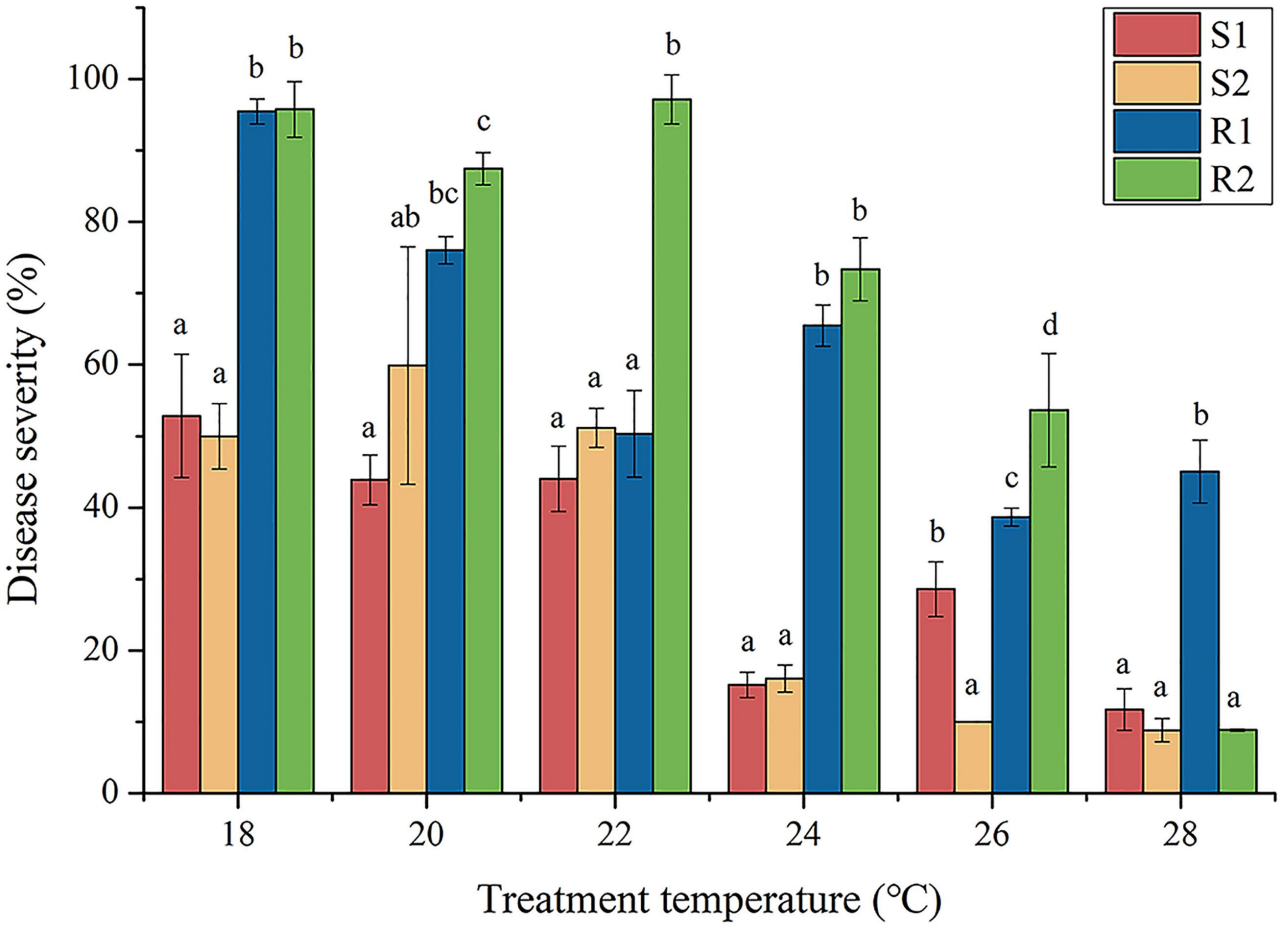
Infection severity of wheat leaf rust with different temperature sensitivity. S1 and S2 were temperature sensitive strains, R1 and R2 were high temperature resistant strains.

According to the analysis in Table 4, the difference between the highest and lowest ET_50_ values of strains from Changzhi was 1.71°C. The highest ET_50_ value of Yuncheng strains was 18.78°C, and the lowest ET_50_ value was 25.50°C, with a difference of 6.72°C. The difference between the highest and lowest ET_50_ values of Jincheng strains was 1.11°C. The difference between the highest and lowest ET_50_ values of Linfen strains was 4.54°C. The difference between the highest and lowest ET_50_ values of Jinzhong strains was 2.49°C. These results indicated that the temperature sensitivity of Yuncheng strains varied significantly compared to other regions. Based on the temperature test results, the disease severity of the four strains (S1, S2, R1 and R2) at different temperature (18°C, 20°C, 22°C, 24°C, 26°C and 28°C) was analyzed using variance analysis (Figure 1). The results showed significantly different in disease severity between high-temperature resistant strains and temperature-sensitive strains at 24°C and 26°C (*p* < 0.05). Notably, at 26°C, there were significant differences in disease severity among all four strains (*p* < 0.05) (Fernandes et al., 2007). To further investigate the effect of high temperature stress on wheat leaf rust, a temperature of 26°C was selected as the optimal condition for high-temperature stress studies.

**Table 4:** Mycelial branch number in four different temperature-sensitive wheat leaf rust at different temperatures.

Strain	T/°C	12 h	24 h	48 h	72 h	120 h
S1	18°C	1.22 ± 0.01a	1.28 ± 0.03c	3.13 ± 0.12b	9.38 ± 0.88b	144.87 ± 24.13ab
	26°C	1.23 ± 0.08a	1.27 ± 0.09c	2.71 ± 0.07b	17.48 ± 1.01a	219.72 ± 6.64a
S2	18°C	1.28 ± 0.08a	2.62 ± 0.15ab	2.81 ± 0.17b	4.17 ± 0.42b	97.38 ± 15.53b
	26°C	1.28 ± 0.10a	2.27 ± 0.11b	2.03 ± 0.07b	3.08 ± 0.29b	117.59 ± 4.57b
R1	18°C	0.98 ± 0.06a	0.88 ± 0.03c	2.32 ± 0.08b	5.15 ± 0.39b	133.48 ± 6.43ab
	26°C	0.97 ± 0.02a	1.35 ± 0.13c	4.33 ± 0.56a	6.75 ± 0.89b	172.22 ± 22.51ab
R2	18°C	1.32 ± 0.05a	2.92 ± 0.18a	2.22 ± 0.08b	8.62 ± 2.53b	172.72 ± 15.90ab
	26°C	1.25 ± 0.04a	2.10 ± 0.22b	2.27 ± 0.20b	3.72 ± 0.57b	174.78 ± 15.19ab

Different letters in the same column represent significant differences between strains at the sametime point and at different temperatures (*p* < 0.05).

### 3.3 Virulence determination and histopathological observation of high-temperature resistant strains and temperature-sensitive strains of *Pt*

**Figure 2:**
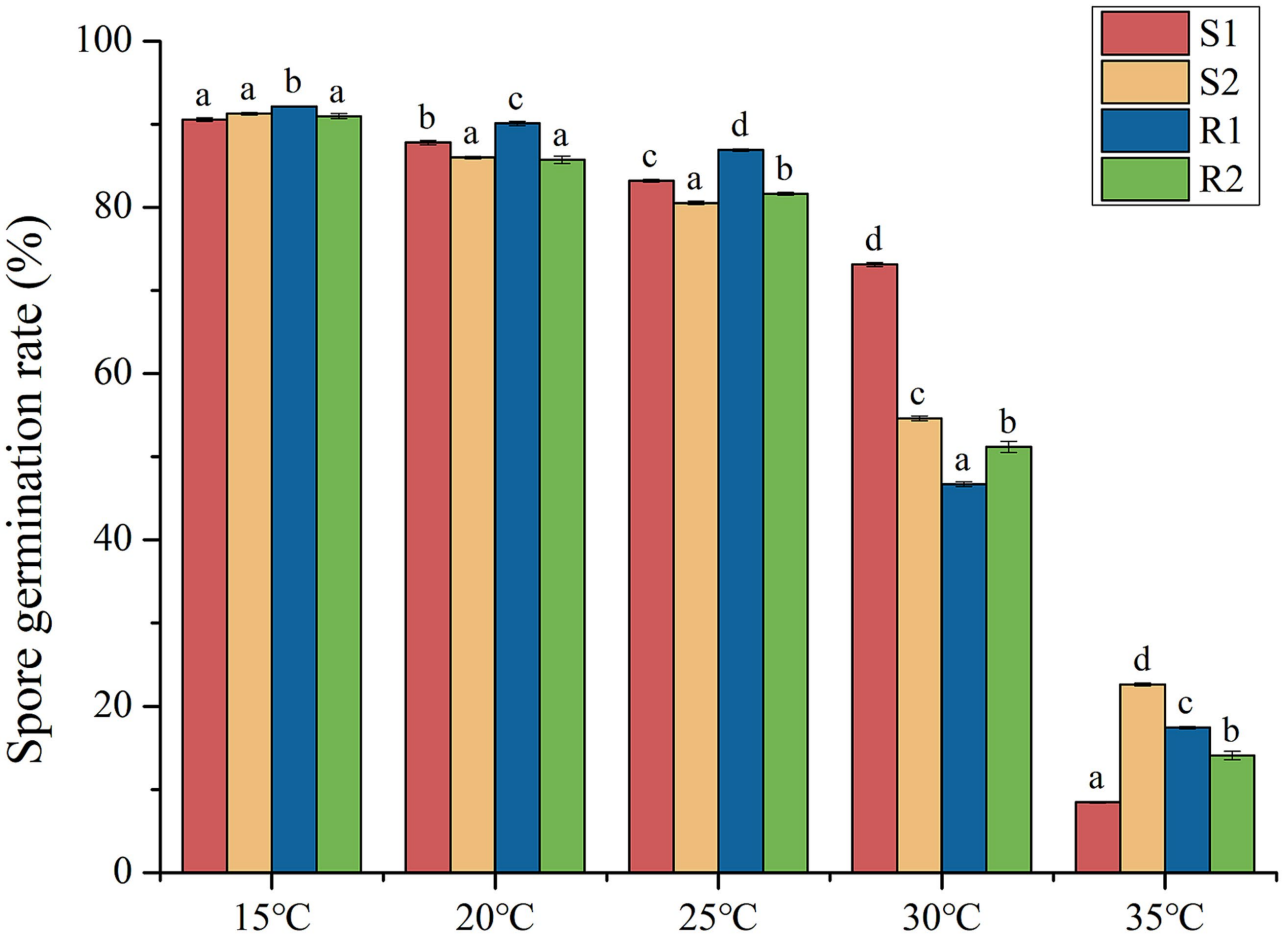
Analysis of variance of urediospores germination rate of strains S1, S2, R1 and R2 at different temperatures.

The germination rates of the four selected strains (S1, S2, R1 and R2) were statistically analyzed, as shown in Figure 2. The results indicated that the germination rate of all four strains was inhibited as the temperature increased. At 15°C, the germination rate of the urediospores for all four strains exceeded 90%, indicating full germination capability under these conditions. Significantly different in germination rates (*p* < 0.05) were observed at 20°C, 25°C, 30°C and 35°C. Notably, the germination rate dropped to less than 25% at 35°C, highlighting the substantial inhibitory effect of higher temperatures on urediospore germination.

**Figure 3:**
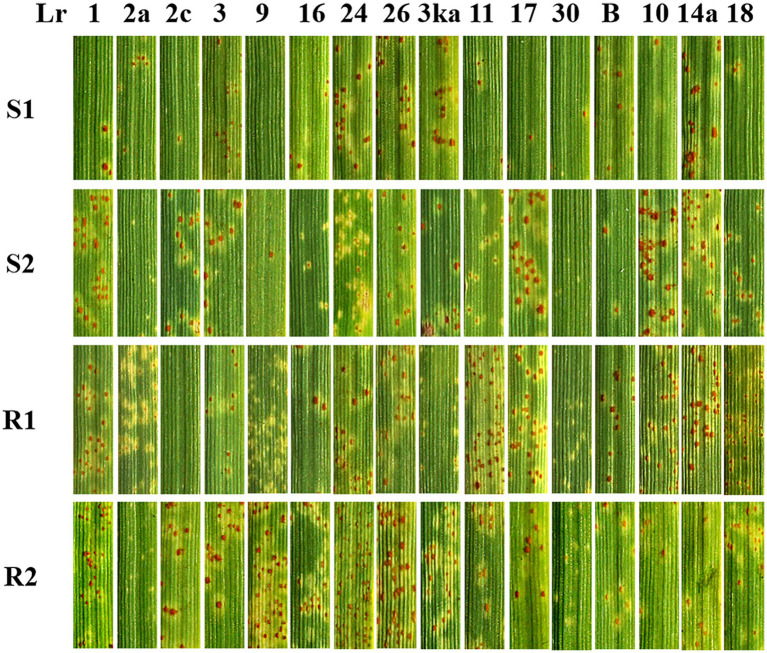
Pathogenic phenotype of test strains on leaf rust resistance near-isogenic lines.

Sixteen near-isogenic lines were used to identify and name the four selected strains. As shown in Figure 3, the pathogenic type of temperature-sensitive strains S1 and S2 were identified as THSJ and RCKK, respectively. Meanwhile, the high-temperature resistant strains R1 and R2 were identified as PKTS and PHSH, respectively.

**Figure 4:**
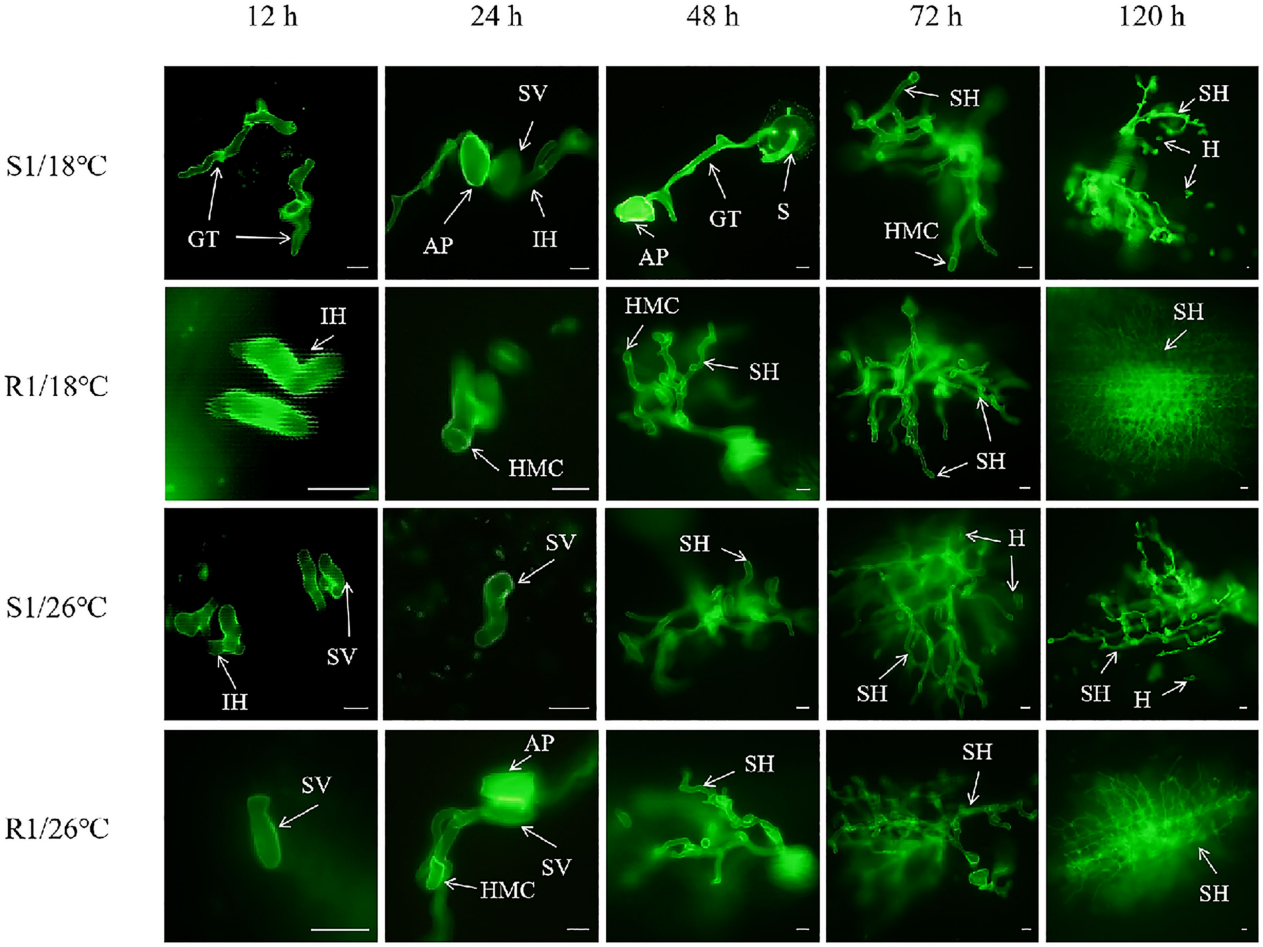
Infection process of wheat leaf rust. Spore (S), germ tubes (GT), Appressorium (AP), substomatal vesicles (SV), infection hyphae (IH), secondary hyphae (SH), haustorial mother cell (HMC), haustoria (H). Bars = 10 μm.

To elucidate the histocytological characteristics of *Pt* at different infection stages, the infection process was observed at various time points post-inoculation. Strains S1 and R1 were used as examples, with results presented in Figure 4. Approximately 12 h after contact with wheat leaves, the urediospores of *Pt* began to germinate, forming germ tubes grew directionally across the leaf surface and extended towards the stomata. Approximately 24 h post-infection (hpi), appressorium formed at the tips of the germ tubes and invaded the stomata. Within the substomatal chambers, irregular substomatal vesicles developed. From the tops of these vesicles, primary infection hyphae began to emerge. Continued growth of the primary infection hyphae led to the differentiation of secondary infection hyphae. By approximately 48 hpi, the apex of the fungus specialized into HMC. These HMCs then formed infection filaments that penetrated mesophyll cells to establism haustoria, thereby completing the formation of the wheat leaf rust infection site. This infection process aligned with previous research findings (Harder and Chong, 1984).

Four strains were inoculated into Mingxian 169 and cultured in incubators at 18°C (control) and 26°C (high temperature stress). SPSS software was used to analyze the histological structure of the infection process for these four strains. The results of mycelial branching number analysis were shown in Table 4. With 12 h after contact with the wheat, all mycelial branches formed, with fewer than two branches observed. This indicated that the urediospores had germinated, formed appressorium and began to differentiate gradually. At 72 hpi, the mycelial branching number of temperature-sensitive strain S1 showed a significant difference between the control group (18°C) and the treatment group (26°C) (*p* < 0.05). In contrast, for the sensitive strain S2, there was no significant difference in the number of mycelium branches between the control group and the treatment groups at any infection times (*p* > 0.05). The mycelium branching number of high-temperature resistant strain R1 showed a significant difference between the control group and the treatment group at 48 hpi (*p* < 0.05). The number of hyphal branches in the control group was 2.32 ± 0.08, while in the treatment group, it reached 4.33 ± 0.56. Similarly, strain R2 exhibited a significant difference at 24 hpi (*p* < 0.05). Compared to the sensitive strain S2, the mycelial branching number of strains S1, R1 and R2 were significantly different under high temperature stress. Overall, there was no significant difference in the number of mycelial branches among the four strains under high temperature stress at different infection times. These results indicated that high temperature stress had minimal impact on the mycelial branching number during the infection process of wheat rust.

The analysis results of the number of HMC were shown in Table 5. HMC formation began at 12 hpi and was most prevalent at 72 h at the majority of infection sites. For strain S1, the number of HMC showed significant differences between the control group and the treatment group at 24 h, 72 h and 120 h (*p* < 0.05), and the strain S2 was significantly different between the control group and the treatment group at 24 hpi (*p* < 0.05). For strain R1, the number of HMC of showed a significant difference between the control group and the treatment group at 48 hpi (*p* < 0.05). The number of haustorium mother cells in the control group was 1.85 ± 0.22, while in the treatment group, it reached 2.47 ± 0.30. In contrast, for strain R2, there were no significant differences in the number of HMC between the control and treatment groups at any infection time (*p* > 0.05). In conclusion, compared to strains S1 and S2, the number of HMC in strains R1 and R2 was less affected by high temperature stress.

**Table 5:** Number of haustorial mother cells in four different temperature-sensitive wheat leaf rust at different temperatures.

Strain	T/°C	12 h	24 h	48 h	72 h	120 h
S1	18°C	0.03 ± 0.03a	0.23 ± 0.05b	1.58 ± 0.10b	4.94 ± 0.54b	118.07 ± 18.08b
	26°C	0.22 ± 0.10a	0.80 ± 0.06a	1.35 ± 0.06b	9.50 ± 0.70a	197.83 ± 2.76a
S2	18°C	0.02 ± 0.01a	0.83 ± 0.16a	0.60 ± 0.09c	0.85 ± 0.22c	71.57 ± 17.72b
	26°C	0.02 ± 0.01a	0.27 ± 0.04b	0.25 ± 0.04c	0.33 ± 0.08c	83.57 ± 2.29b
R1	18°C	0.00 ± 0.00a	0.43 ± 0.03ab	1.85 ± 0.22b	3.38 ± 0.20b	105.22 ± 4.41b
	26°C	0.11 ± 0.00a	0.72 ± 0.15a	2.47 ± 0.30a	4.02 ± 0.40b	134.52 ± 19.80b
R2	18°C	0.02 ± 0.01a	0.46 ± 0.04ab	0.40 ± 0.08c	1.42 ± 0.30c	112.22 ± 1.15b
	26°C	0.03 ± 0.03a	0.25 ± 0.02b	0.50 ± 0.14c	1.28 ± 0.05c	105.32 ± 9.74b

Different letters in the same column represent significant differences between strains at the sametime point and at different temperatures (*p* < 0.05).

The analysis results for the number of H were shown in Table 6. H began to form successively after 24 hpi and were most prevalent at 72 h at majority of infection sites. The number of H for strain S1 showed significant differences between the control group and the treatment group at 72 hpi and 120 hpi (*p* < 0.05). In contrast, for strain S2, significant differences were observed only at 24 hpi (*p* < 0.05). The number of haustoria in the control group was 0.48 ± 0.14, while in the treatment group, it reached 0.15 ± 0.02. For strains R1 and R2, there were no significant differences in the number of H between the control group and the treatment group at any time point (*p* > 0.05). In summary, compared to strains S1 and S2, the number of H of strains R1 and R2 was less affected by high temperature stress.

**Table 6:** Number of haustoria in four different temperature-sensitive wheat leaf rust at different temperatures.

Strain	T/°C	12 h	24 h	48 h	72 h	120 h
S1	18°C	0.02 ± 0.01a	0.02 ± 0.01b	0.65 ± 0.06a	1.70 ± 0.27b	90.57 ± 14.50b
	26°C	0.00 ± 0.00a	0.03 ± 0.03b	0.33 ± 0.05ab	4.23 ± 0.20a	166.08 ± 5.68a
S2	18°C	0.02 ± 0.01a	0.48 ± 0.14a	0.37 ± 0.04ab	0.37 ± 0.10 cd	53.97 ± 13.87b
	26°C	0.00 ± 0.00a	0.15 ± 0.02b	0.07 ± 0.01b	0.18 ± 0.05d	50.58 ± 2.74b
R1	18°C	0.00 ± 0.00a	0.00 ± 0.00b	0.65 ± 0.10a	1.12 ± 0.07bc	71.40 ± 2.38b
	26°C	0.00 ± 0.00a	0.02 ± 0.01b	0.73 ± 0.21a	1.65 ± 0.31b	101.62 ± 17.33b
R2	18°C	0.00 ± 0.00a	0.28 ± 0.01b	0.10 ± 0.00b	0.53 ± 0.08 cd	81.32 ± 2.27b
	26°C	0.02 ± 0.01a	0.07 ± 0.04b	0.32 ± 0.12ab	0.78 ± 0.05 cd	67.45 ± 4.73b

Different letters in the same column represent significant differences between strains at the same time point and at different temperatures (*p* < 0.05).

In summary, the analysis revealed that the number of HMC and H in the high-temperature resistant resistant strains R1 and R2 was generally less affected by high temperature stress. This contrasts with the significant impacts observed in the sensitive strains S1 and S2, highlighting the differential response to elevated.

### 3.4 *Hsp* genes expression of *Pt* under high temperature stress

To investigate the molecular response to high temperature stress, the expression levels of heat shock proteins (HSP90-1, Hp, HSPHSS1, HSP60 and HSPSSB) were analyzed in the four strains (S1, S2, R1 and R2) at different time points. This analysis aimed to elucidate how these key genes, which play critical roles in stress response and protein homeostasis, were regulated under elevated temperatures. The results offered valuable insights into the differential gene expression patterns among the strains, highlighting potential mechanisms of thermal resistance.

**Figure 5:**
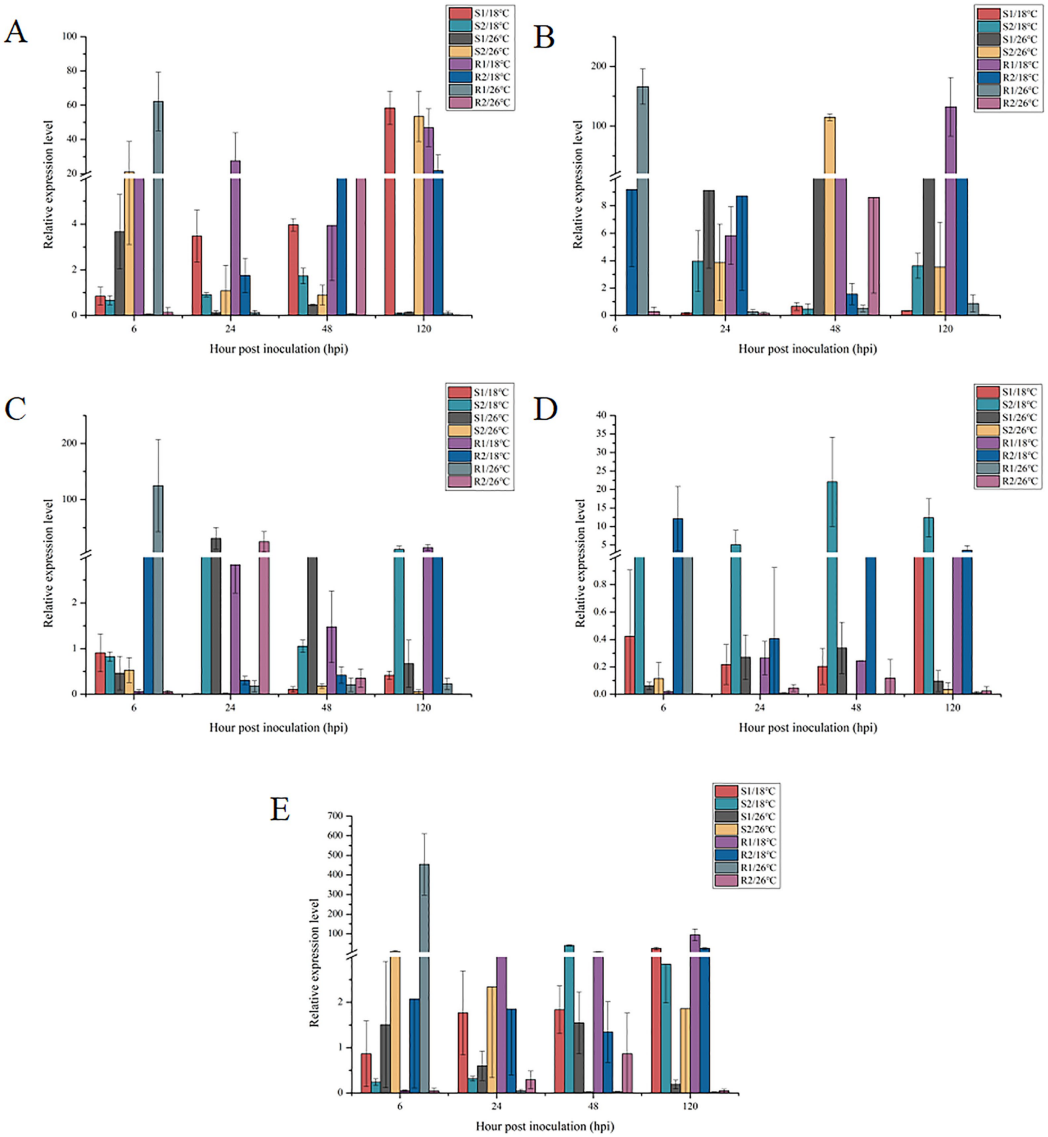
Expression of *Pthsp* genes in *Puccinia triticina* with different temperature sensitivity under high temperature stress. S/18°C: sensitive strains treated at 18°C; R/18°C: high temperature resistant strains treated at 18°C; S/26°C: sensitive strains treated at 26°C; R/26°C: high temperature resistant strains treated at 26°C; **A–E** refers to *Pthsp90-1*, *Pthp*, *PthspHSS1*, *Pthsp60* and *PthspSSB* genes, respectively.

As shown in Figure 5, the expression of the *Pthsp90-1* gene varied significantly with infection time and temperature conditions. For strains S1 and R2 at 18°C, *Pthsp90-1* gene expression increased over time, reaching its maximum value at 120 hpi. In contrast, at 26°C, the expression in strains S1 and R1 initially peaked at 6 hpi but then gradually decreased. For strain R1 at 18°C, *Pthp* gene expression increased gradually, reaching its maximum at 120 hpi. For strain R2 at 26°C, *Pthp* gene expression initially increased and then decreased, peaking at 48 hpi. For strain R2 at 18°C, *Hp* gene expression initially decreased and then increased. The expression reached its minimum value at 48 hpi. For strain R1 at 26°C, *Pthp* gene expression initially decreased, reaching its maximum value at 24 hpi. It then increased, peaking at 6 hpi. The expression of *Pthp* gene in strain S1 remained relatively stable at both 18°C and 26°C, with no significant trend in change observed. Notably, at 18°C, the expression level was low and exhibited minimal variation. The expression of the *PthspHSS1* gene in strain R1 gradually decreased at 26°C, reaching its maximum value at 6 hpi. In contrast, at 18°C, the expression pattern for the *PthspHSS1* gene in strain S1 and R2 initially declined, hitting its minimum value was reached at 24 hpi, before subsequently increasing to reach a peak at 6 hpi. The expression of the *PthspHSS1* gene in strain R1 gradually decreased at 26°C, peaking at 6 hpi. The expression of the *Pthsp60* gene in strain R1 at 26°C showed a gradual decrease, yet it reached its maximum level 6 hpi. In contrast, the *Pthsp60* gene expression in strain S2 at 18°C and in strain S1 and R2 at 26°C followed a similar trend. Initially, expression increased before subsequently decreasing, with all three strains reaching their maximum expression levels 48 hpi. With extended infection time, the *PthspSSB* gene expression in strains S1 and R1 at 18°C gradually increased, peaking at 120 hpi. In contrast, at 26°C, the *PthspSSB* gene expression in strain R1 gradually decreased, reaching its maximum at 6 hpi. For strain S2 at 18°C and strain R2 at 26°C, the *PthspSSB* gene expression exhibited a similar trend: it initially increased before decreasing, with the peak expression occurring 48 hpi. Conversely, the *PthspSSB* gene expression in strain S2 at 26°C and strain R2 at 18°C showed an opposite pattern, initially decreasing before increasing, and reaching its minimum at 48 hpi.

## 4 Discussion

Environmental conditions were fundamental factors influencing the occurrence and development of plant diseases. Global warming significantly impacts both plant growth and disease dynamics. On the one hand, rising temperatures expand the geographical distribution of pathogens and increase their population sizes. On the other hand, global warming fostered the emergence of heat-tolerant pathogen strains and prolonged their survival time, potentially exacerbationg disease incidence.

In this study, we assessed the temperature sensitivity of 41 strains of wheat leaf rust isolated from various cities in Shanxi Province. We examined the biological characteristics and expression of *HSP* genes in two high-temperature resistant strains and two temperature-sensitive strains under high temperature stress. Our findings revealed that the average ET_50_ for the 41 tested strains was 22.81°C, with the highest ET_50_ value being 22.50°C and lowest at 18.78°C, resulting in a range of 6.72°C. Notably, both the strains with the highest ET_50_ and the one with lowest ET_50_ in 2021–2022 were collected from Yuncheng city, located in the southernmost part of Shanxi Province. This region was a significant wheat-growing area within Shanxi and falled within the Jinshanyu Yellow river “Golden Triangle.” The concentration of extreme ET_50_ values in Yuncheng City may be attributed to its unique geographical location and climatic conditions, which could influence the selection and survival of specific rust pathogen strains (Li H. F. et al., 2023; Li H. H. et al., 2023).

Henan Province, the largest wheat-producing region in china, and Shaanxi Province, another significant wheat-growing area, have both been impacted by outbreaks of wheat leaf rust. These regions played crucial roles in national wheat production, marking the management and control of wheat leaf rust essential to maintaining agricultural productivity and food security. The diversity of leaf rust varieties in Yuncheng was notably abundant, potentially influenced by air flow patterns, which may contribute to the wide range of ET_50_ values observed among the leaf rust strains. During the 2021–2022 period, samples of wheat leaf rust were collected from five key cities in Shanxi Province: Changzhi, Yuncheng, Jincheng, Linfen and Jinzhong, These locations collectively covered the major wheat-growing regions of Shanxi Province, providing a comprehensive overview of the disease’s distribution and variability within the province. The ET_50_ values, representing the temperature sensitivity of *Pt* strains, varied significantly across different regions, indicating that the temperature sensitivity of *Pt* strains differed not only between but also within wheat-growing regions under natural conditions. This variability suggested that *Pt* strains from different wheat areas, or even from the same region, may originate from the same or adjacent fungal source centers and over-summering areas. However, their temperature sensitivity can differ due to local climatic conditions and geographic distance. These findings were consistent with previous studies on temperature sensitivity in wheat pathogens. Specifically, they aligned with the results reported by Ming et al. (2012) for wheat powdery mildew and Lian et al. (2016) for wheat stripe rust, highlighting the influence of environmental factors on pathogen behavior and distribution.

The virulence identification results of four strains in this study (Figure 3) showed that their pathogenic types were THSJ (S1), RCKK (S2), PKTS (R1), and PHSH (R2). Previous studies reported that the main pathogenic types of leaf rust fungus strains collected from Jiangsu, Zhejiang, and Anhui provinces during 2019–2020 were THS, PHS, SHJ, and SHS (Li H. F. et al., 2023; Li H. H. et al., 2023). This indicated that the four strains selected belonged to the currently prevalent dominant races. Current research on the virulence analysis of wheat leaf rust primarily focuses on major wheat-producing regions such as Henan, Shandong, Sichuan, Hebei, Hubei, Xinjiang, and Guizhou provinces (Xia et al., 2023; Xu et al., 2023). However, since wheat leaf rust was an air-transmitted disease, understanding the pathogenic types of wheat leaf rust in southern Shanxi, which served as a critical summer-survival area for the pathogen, was essential.

To effectively prevent and control the occurrence and spread of wheat leaf rust, it is crucial to analyze the biological characteristics and gene expression of different temperature-sensitive strains. In this experiment, we assessed disease severity under various temperature conditions to determine the optimal temperature for leaf rust strain growth under high-temperature stress (Zhang et al., 2022). This approach provided valuable insights into how temperature influences pathogen behavior, aiding in the development of targeted management strategies. From Figure 1, it was evident that there were significant differences in disease severity between strains R1 and R2 (*p* < 0.05). In contrast, strain R1 did not show significant differences in disease severity when compared to strains S1 and S2 (*p* > 0.05). Additionally, with the stress temperature increased, significant differences in disease severity were observed among strains R1, S1 and S2 (*p* < 0.05). At 28°C, strain R2 did not show significant differences in disease severity compared to strains S1 and S2 (*p* > 0.05). This contrasted with strain R1, which exhibited significantly lower disease severity under the same conditions, indicating that strain R1 has a higher tolerance to high temperature than strain R2. Therefore, it can be inferred that both strain R1 and R2 were high-temperature resistant strains, but R1 demonstrated greater resistance to high temperature than R2. Shin et al. (2018) demonstrated that strains HTR-1 and HTR-2 of *Pyropia yezoensis* exhibited resistance to high temperature at 20°C, with strain HTR-1 showing greater resistance compared to HTR-2. This conclusion was similar to our experimental results, where strain R1displayed significantly higher tolerance to elevated temperatures than strain R2. Both studies highlighted the variability in high-temperature resistance among different strains, underscoring the importance of strain-specific characteristics in responding to thermal stress.

The germination rate of urediospores and the histological structure of four wheat leaf rust strains were evaluated at different temperatures. The results indicated that the germination rate of urediospores was inhibited as temperature increased, with an optimal germination temperature of was 15°C. The optimal temperature for wheat leaf rust urediospores was higher than that reported for wheat stripe rust (Cheng et al., 2014), and the germination speed was relatively rapid, suggesting a preference for slightly higher temperature. Consequently, rising global temperatures have extended the prevalence period of wheat leaf rust, potentially leading to greater harm to wheat yield and quality. The prolonged exposure to favorable temperatures may enhance the spread and impact of this pathogen, underscoring the need for adaptive agricultural practices and disease management strategies.

By observing the infection process under high temperatures, we gained further insights into the histological structure and biological characteristic of different temperature-sensitive strains. Our findings indicated that high temperatures can inhibit the expansion of wheat leaf rust to a certain extent, a phenomenon also observed in other fungi subjected to abiotic stress (Zhao et al., 2019). Specifically, high temperatures inhibited the expression of cell cycle-related genes and interfered with conidia germination and mycelium growth. Interestingly, when comparing temperature-sensitive strains to high-temperature resistant strains, we found that high-temperature stress had no significant effect on the number of HMC and H during the 0–120 h infection period in high-temperature resistant strains. This suggests that formation of HMC and H formation at the late stage of infection were critical for resistance to heat stress. Consistent with these findings, Zhang et al. (2022) reported that formation of H and mycelium expansion in wheat powdery mildew were the key infection stages where high-temperature resistant strains demonstrated resistance to heat stress. However, the mechanisms by which high-temperature resistant strains confer this resistance remained unclear. Further experimental studies were necessary to elucidate how these strains maintain their resistance during critical infection stages and to uncover the underlying mechanisms involved.

In this study, to identify *HSP* genes in the wheat leaf rust, we utilized the amino acid sequences of HSP83, HSPHSS1, HSP60, HSPSSB, and DnaJC19 (HSP40) from *Puccinia graminis* f. sp. *tritici* as query sequences. These sequences were used to search the *Pt* genome under the NCBI accession GCA_026914185.1. In this study, we investigate the high-temperature resistance mechanism of wheat leaf rust by measuring the relative expression levels of HSP genes. Future experimental plans may involve genomic sequencing of different strains, followed by GWAS (Genome-Wide Association Study) analysis to identify SNP (Single Nucleotide Polymorphism) sites associated with high-temperature resistance (Yang et al., 2019). Additionally, current research focuses more on gene products. Therefore, using metabolomics techniques to screen for key metabolites was crucial for further identifying their functions (Zhang et al., 2024) and metabolic pathways (Wang et al., 2024).

The results showed that compared to 18°C, the expression of the *Pthsp90-1* gene in strain S1 was down-regulated at 26°C after 120 h. Conversely, for strain R1, the expression of *Pthsp90-1* gene was up-regulated at 26°C after just 6 h. HSP90 was a eukaryotic chaperone that was expressed typically expressed in large quantities in response to rising temperatures (Dal Piaz et al., 2015). For instance, the expression of *Ichsp90* peaked at 39°C, reaching levels 5.02 times higher than those of control samples, underscoring its critical role in resisting external temperature stress (Wang et al., 2016). In studies on *Aspergillus fumigatus*, HSP90 has been shown to play a crucial role in spore growth and cell wall formation (Lamoth et al., 2012). Similarly, Yang et al. (2006) found that HSP90 was involved in the hyperosmotic stress response in *Saccharomyces cerevisiae*. Additionally, Park et al. (2014) demonstrated that *HSP90-1* played an important role in agrobacterium-mediated transformation. The induction of *HSP90*-1 by high-temperature suggested its involvement in defense against high temperature stress. In conclusion, *Pthsp90*-1 likely participated in resistance to high temperature stress during the germination stage of *Pt* urediospores. Located in the cytoplasm, *Pthsp90*-1 may be involved in cell growth and other processes. However, the specific mechanisms by which it contributed to heat stress resistance remain to be further investigated.

Compared to 18°C, the expression of the *PthspHSS1* gene in strain R1 was up-regulated at 26°C for 6 h. This suggested that *PthspHSS1* also played an important role in the germination stage of *Pt* urediospores, aiding in resistance to high temperature stress. Similar observations have been made with other heat shock proteins (HSPs) under abiotic stress conditions. For instance, when *Trichoderma* was grown at 37°C or 41°C in the presense of oxiants or penetrants, *HSP70* gene expression was up-regulated, indicating that HSP70 proteins enhance tolerance to abiotic stress. In *Fusarium pseudograminearum*, it was found that *Fphsp70* may play a key role in pathogenicity. Knocking out the *HSP70* homologous gene *FpLhs1*, located in the endoplasmic reticulum, resulted in inhibited growth, reduced conidiation, and weakened pathogenicity of F.*pseudograminearum* (Chen et al., 2019). Batcho et al. (2021) also reported that, with increasing heat stress duration, the relative expression of *AsHSP70* in transgenic plants induced by polyamine oxidase increased from 1.02 to 9.58. Given that *PthspHSS1* was located in the cytoplasm, it likely participated in cell growth and other processes, contributing to high temperature stress resistance during the critical germination stage of urediospores. However, further studies were needed to elucidate the specific mechanisms by which *PthspHSS1* confers this resistance.

The analysis of *Pthsp60* gene expression revealed that, compared to 26°C, strain S2 showed significant up-regulation at 18°C after 48 h and 120 h, while strain R2 exhibited significant up-regulated at 18°C after just 6 h. These finding suggested that lower temperature enhance *Pthsp60* gene expression in these strains during critical infection stages. HSP60 has been identified as playing a crucial role in stress response. In pepper, knocked out the *CaHsp60*-6 gene increased sensitivity to heat stress, leading researchers to speculate that HSP60 may be a key gene in defending against heat stress and other abiotic stresses (Ul Haq et al., 2019). The result from this study were consistent with those findings, further supporting the inference that HSP60, particularly when localized in mitochondria, played an important role in resisting high temperature stress during the germination stage of *Pt* urediospores.

Analysis of the relative expression level of *PthspSSB* gene revealed that, compared to 18°C, strain R1 showed significant up-regulation after infection at 26°C for 6 h. HSPSSB, identified as a cytoplasmic chaperone similar to HSP80, appears to play a critical role in thermal stress responses. Zhu et al. (2023) demonstrated that *StHsfB*, a heat shock transcription factors in potatoes, acted as a coactivator enhancing by directly inducing the expression of target genes such as *sHsp17*.6, *sHsp21*, *sHsp22*.7 and *Hsp80*. Thisfinding suggested that heat shock proteins, including Hsp80-like *PthspSSB*, were crucial for mitigating the effects of high temperatures. Given these insights, it can be concluded that the *PthspSSB* gene likely played a vital role during the germination stage of *Pt* urediospores. Its overexpression may reduce protein misfolding, thereby contributing significantly to wheat’s resistance to high temperature stress. The enhanced expression of under elevated temperatures supported its function in protecting cellular processes and structures against thermal damage, potentially improving the pathogen’s survival and infectivity under adverse conditions.

From the above findings, it can be inferred that *Pthsp90-1*, *Pthp*, *PthspHSS1* and *Pthsp60* were significantly up-regulated at 6 hpi. This suggested that these HSPs played an important role during the early germination stage of *Pt* urediospores, aiding in their resistance to heat stress. Kumar et al. (2021) found that microRNAs (miRs) regulated the expression of heat shock proteins (HSPs)/ heat shock transcription factors (HSFs) during the initial stages of heat stress, promoting plant survival and development under high temperature. Similarly, extreme heat stress induced the accumulation of *Hsp70* and *Hsp17*.6 genes in soybean seeds, as reported by Krishnan et al. (2020). These studies underscored the importance of HSPs in protecting cellular functions and structures against heat stress.

In the future, it will be crucial to continue monitoring the changes in temperature sensitivity of *Pt* across different areas of Shanxi Province. In summary, expanding the monitoring scope was essential for advancing our understanding of how wheat leaf rust populations adapt to temperature changes. This approach will not only enhance our knowledge of pathogen behavior but also inform effective, region-specific strategies to protect wheat crops from this significant threat.

## Data Availability

The original contributions presented in the study are included in the article/Supplementary material, further inquiries can be directed to the corresponding author.

## Supplementary material

SUPPLEMENTARY TABLE 1 Sequences of the primers for determining the mRNA relative expression of *Pt*.
